# Analysis of cytokine levels, cytological findings, and MP‐DNA level in bronchoalveolar lavage fluid of children with *Mycoplasma pneumoniae* pneumonia

**DOI:** 10.1002/iid3.849

**Published:** 2023-05-08

**Authors:** Fang Deng, Huiling Cao, Xiaohua Liang, Qubei Li, Yang Yang, Zhihua Zhao, Junjie Tan, Guo Fu, Chang Shu

**Affiliations:** ^1^ Department of Respiratory, Children's Hospital of Chongqing Medical University, National Clinical Research Center for Child Health and Disorders, Ministry of Education Key Laboratory of Child Development and Disorders, China International Science and Technology Cooperation base of Child development and Critical Disorders Chongqing Key Laboratory of Pediatrics Chongqing China; ^2^ Department of Pediatrics Affiliated Hospital of North Sichuan Medical College Nanchong China; ^3^ Department of Neonatology Children's Hospital of Chongqing Medical University Chongqing China; ^4^ Department of Clinical Epidemiology and Biostatistics Children's Hospital of Chongqing Medical University Chongqing China; ^5^ Clinical Molecular Medical Center Children's Hospital of Chongqing Medical University Chongqing China

**Keywords:** bronchoalveolar lavage fluid, cytokines, cytology, DNA, *Mycoplasma pneumoniae* pneumonia

## Abstract

**Background:**

The present study was conducted to determine the inflammatory response in the lungs of children with *Mycoplasma pneumoniae* pneumonia (MPP).

**Methods:**

This study retrospectively analyzed cytokine levels, cytological findings, and *M. pneumoniae* (MP)‐DNA level in the bronchoalveolar lavage fluid (BALF) of 96 children with MPP. The study utilized Spearman's correlation method to evaluate the contribution of BALF and blood parameters in MPP children.

**Results:**

(1) A total of 96 MPP children were classified into the Low MP‐DNA MPP group (BALF MP‐DNA ≤ 10^5^ copies/mL) and the High MP‐DNA MPP group (BALF MP‐DNA > 10^5^copies/mL); the Non‐fever MPP group (no fever during the entire course of pneumonia) and the Fever MPP group; the Defervescence MPP group (fever had subsided at the time of bronchoscopy) and the Fervescence MPP group; and the Mild MPP group and the Severe MPP group. (2) The High MP‐DNA MPP, Fever MPP, Fervescence MPP, and Severe MPP groups had higher levels of interleukin (IL)‐6, IL‐10, and tumor necrosis factor‐α (TNF‐α) in their BALF (all *p* < .05). (3) The proportions of neutrophils and macrophages in the BALF of the High MP‐DNA MPP and Fever MPP groups increased and decreased, respectively (all *p* < .05). (4) In the BALF of MPP children, MP‐DNA, IL‐6, IL‐10, TNF‐α, and interferon gamma (IFN‐γ) levels positively correlated with neutrophil proportion while negatively correlated with macrophage proportion (all *p* < .05). (5) The MP‐DNA, IL‐6, IL‐10, TNF‐α, and IFN‐γ levels in the BALF of MPP children were positively correlated with the levels of C‐reactive protein, procalcitonin, lactic dehydrogenase, fibrinogen, and d‐dimer, while they were negatively correlated with the albumin level (all *p* < .05).

**Conclusions:**

In children with MPP, the pulmonary inflammatory immune response was stronger in the High MP‐DNA MPP, Fever MPP, Fervescence MPP, and Severe MPP groups. The relationship between pulmonary cytokine levels, MP‐DNA load, and serum inflammatory parameters were found to be weak.

## Graphical abstract

In children with MPP, the pulmonary inflammatory immune response was stronger in the High MP‐DNA MPP, Fever MPP, Fervescence MPP, and Severe MPP groups. The relationship between pulmonary cytokine levels, MP‐DNA load, and serum inflammatory parameters was found to be weak.

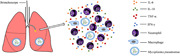

## 1 INTRODUCTION

*Mycoplasma pneumoniae* (MP) infection is common in preschool and school‐age children. *M. pneumoniae* pneumonia (MPP) accounts for approximately 40% of cases of community‐acquired pneumonia.^1^ MP causes disease directly through its toxic effects on alveolar epithelial cells^2^ or indirectly through immune disorders caused by infection.^3^ Apart from inducing pathological alterations in the respiratory tract, MP infection has the potential to impact systemic organs. Furthermore, severe MPP (SMPP, refers to MPP that meets the criteria for severe pneumonia^4^) or refractory MPP (RMPP, refers to MPP i.e., prolonged or has worsened clinical and/or imaging course despite 7 days of reasonable treatment with macrolide antibiotics^5^) can lead to bronchiolitis obliterans, leading to a long‐term impairment of lung function.^4^

MPP is usually treated with macrolide antibiotics.^2^ However, some children with MPP do not respond to macrolides in the first three days of treatment (macrolide nonresponsive MPP).^5^ Recently, MP infection has become progressively more common in younger age groups.^5^ Furthermore, the incidence of macrolide‐resistant MPP is gradually increasing each year.^6^ Over the last 20 years, the prevalence of macrolide‐resistant MP in Asia has surpassed 60%, and in China and Korea, it has surpassed 80%.^7^ Therefore, there is an urgent need to consider alternative antibiotics for treating MPP.

Previous studies^8^, ^9^ have reported that large amounts of cytokines are released in the blood of patients with SMPP and RMPP. Cytokines play an important role in mediating the immune inflammatory response.^10^ Microbial infections trigger an exaggerated activation of immune cells that release a vast quantity of cytokines within a brief period, culminating in a cytokine storm.^4^ The more severe the clinical disease and organ damage, the stronger the immunoinflammatory response and cytokine activation.^5^ Immunotherapy is an important treatment approach to improve the condition and outcome of inflammatory diseases.^11^

Previously, we discovered differences in the cytokine profiles of children with MPP in their lungs and blood (see Figure S1). We posit that the affected lung marks the initiation of an inflammatory response and immune dysregulation. Therefore, the immune‐inflammatory response in the lungs of pediatric patients with MPP warrants the attention of clinicians. This information may assist clinicians in determining the necessity of initiating immunotherapy in children diagnosed with MPP. Therefore, the present study retrospectively analyzed the levels of cytokines (IL‐2, IL‐4, IL‐6, IL‐10, IL‐17A, TNF‐α, and IFN‐γ), cytological findings, and MP‐DNA level in bronchoalveolar lavage fluid (BALF) of children with MPP to provide more information for initiating immunotherapy in children with MPP.

## 2 MATERIALS AND METHODS

### 2.1 Patients

This was a retrospective observational study. This study collected medical records and laboratory data of pediatric patients diagnosed with MPP, who met the inclusion criteria and were admitted to the Children's Hospital between March 2019 and February 2020. The inclusion criteria were as follows: (1) met the diagnostic criteria for MPP; MPP was diagnosed based on the presence of respiratory symptoms, with or without fever, lung inflammation confirmed by chest X‐ray or CT scan, positive MP‐DNA in nasopharyngeal aspirates (NPAs) and/or BALF, and serum MP‐specific IgM antibodies ≥ 1:160 and/or IgG antibodies ≥ 4‐fold increase^4^; (2) underwent bronchoscopy and provided BALF sample for cytokine, cytological, and etiological testing; and (3) aged 29 days to below 18 years.

The following were the exclusion criteria: (1) children with asthma, tuberculosis, or other respiratory diseases; (2) children with severe diseases of important organs, tumors, immunological diseases, or malnutrition before admission; (3) children receiving immunosuppressants or glucocorticoids before admission; (4) children in whom pathogens other than MP or both MP and other etiological agents were detected in NPAs, sputum culture, or BALF; and (5) children who did not have a bronchoscopy and were unable to provide BALF for cytokine, cytological, and etiological testing.

Bronchoscopy was performed on MPP children in the following situations^12^: (1) children with wheezing, recurrent or persistent, or localized, (2) children with recurrent respiratory tract infections, (3) children suspected to have foreign body aspiration, (4) children who experienced difficulty in ventilator withdrawal, (5) children with abnormal chest imaging findings: [a] pulmonary airway malformation; [b] atelectasis; [c] emphysema; [d] dysplasia of blood vessels, lymphatic vessels, or esophagus; and [e] pleural effusion requiring differential diagnosis, (6) children who required etiological diagnosis and treatment, and (7) other conditions that required bronchoscopy as assessed by the pediatrician.

A total of 96 MPP children are divided into the following groups: 1) according to the MP‐DNA load, the children were classified into the Low MP‐DNA MPP group (MP‐DNA ≤ 10^5^ copies/mL, *n* = 28) and the High MP‐DNA MPP group (MP‐DNA > 10^5^ copies/mL, *n* = 68); 2) based on whether they had a fever during the entire course of pneumonia, the children were classified into the Non‐fever MPP group (children who did not have a fever during the whole period of pneumonia, *n* = 14) and the Fever MPP group (*n* = 82); furthermore, the Fever MPP group of 82 children was further subdivided into the Defervescence MPP group (children whose fever had subsided at the time of bronchoscopy, *n* = 65) and the Fervescence MPP group (children who still had fever at the time of bronchoscopy, *n* = 17), based on their fever at the time of bronchoscopy; 3) based on the severity of pneumonia (see Table S1), the children were classified into the Mild MPP group (*n* = 37) and the Severe MPP group (*n* = 59).

The present retrospective observational study was authorized by the Medical Research Ethics Committee of the Children's Hospital of Chongqing Medical University and registered at http://www.chictr.org.cn/with the registration number ChiCTR2000034048 (registration date: June 22, 2020). Data for this study were sourced from medical records and laboratory findings from prior clinical consultations. The requirement to obtain informed consent was waived.

### 2.2 Bronchoscopy

Bronchoscopy indications and procedures followed the Chinese pediatric flexible bronchoscopy guidelines (2018 version).^12^ Transnasally, an electronic bronchoscope was inserted, and each segment of the bronchus was examined sequentially. The findings of preoperative chest imaging were used to localize. The bronchoscope was inserted into the target bronchus, and 37°C saline lavage (1 mL/kg, ≤20 mL each time) was given, followed by negative pressure suction to obtain BALF. The obtained BALF samples were used to perform cytokine, cytological, and etiological assays.

### 2.3 Flow cytometric assay

The levels of cytokines (IL‐2, IL‐4, IL‐6, IL‐10, IL‐17A, TNF‐α, and IFN‐γ) were determined by flow cytometric beard assay using commercial human Th1/Th2/Th17 test kits (SaiKi Biotechnology, Jiangxi, China). The lowest detection limit (LODL) of the cytokines was 2.5 pg/mL. The upper limits of the normal reference values of IL‐2, IL‐4, IL‐6, IL‐10, IL‐17A, TNF‐α, and IFN‐γ were 9.80, 3.00, 16.60, 4.90, 14.80, 5.20, and 17.30 pg/mL, respectively.

### 2.4 BALF cytology

Cytological examination of the BALF was performed by the clinical laboratory at the Children's Hospital. Mucus in the BALF sample was first removed. The pretreated BALF samples were mixed with 0.2% trypan blue solution at the ratio of 1:1, and 20 µL of the mixture was filled into a counting plate for cell counting using the Counstar automated cell counter (Ruiyu Biotech). A laboratory centrifuge (Thermo Fisher Scientific, Shanghai, China) was employed to process the BALF samples, and the supernatant was discarded. Subsequently, Wright–Giemsa stain (Baso Diagnostic Ltd.) was utilized for staining purposes. Light microscopy was used for cell sorting and counting at a magnification of 40×.

### 2.5 qRT‐PCR

MP nucleic acids were determined by the SLAN‐96P real‐time polymerase chain reaction (qRT‐PCR) instrument (Shanghai Hongshi) using the *M. pneumoniae* DNA fluorescence diagnostic kit (Sansure Biotech).

### 2.6 Passive particle agglutination

Serum MP‐specific antibodies were measured through passive particle agglutination using an antibody detection diagnostic kit (SERODIA‐MYCO II).

### 2.7 Statistical analysis

Statistical software SPSS version 25.0 and GraphPad Prism version 8.0.1 were used for data analysis. Data exceeding the LODL were represented as ½ of LODL for statistical processing. Non‐normally distributed data were represented as median (interquartile range), while normally distributed data were presented as mean ± standard deviation. An unpaired *t*‐test was used to compare the normally distributed parameters of the two groups. The Mann–Whitney *U* test was employed for nonparametric variables. The Chi‐square or Fisher's exact test was utilized for classified variables. The role of BALF cytokines, inflammatory cells, and MP‐DNA level in children with MPP was investigated using Spearman's correlation. The correlation between the parameters was examined using Spearman's correlation coefficient (*r*). A two‐tailed *p*‐value of < .05 was considered to be statistically significant.

## 3 RESULTS

### 3.1 Patient characteristics

The clinical and laboratory data collected from the 96 children with MPP are shown in Tables 1 and 2, respectively. The study cohort included 51 boys and 45 girls, with a mean MP‐DNA level of 10^6^ copies/mL (range: 10^5^–10^8^ copies/mL). Five children received no antibiotics, 40 received only macrolide antibiotics, one child received only nonmacrolide antibiotics, and 50 received both macrolides and non‐macrolides. Nine children were treated with immunotherapy (three with glucocorticoids 1–2 mg/kg/d, four with immunoglobulins 400–500 mg/kg/d, and two with both these approaches).

**Table 1:** Patient characteristics, pulmonary and extrapulmonary manifestations of 96 children with MPP.

	MPP children (*n* = 96)
Male [*N*(%)]	51 (53.13)
Age [$\bar{x}$ ±s, years]	5.36 ± 2.73
Weight [M(P_25_–P_75_), kg]	18.00 (14.00–23.00)
Fever days [M(P_25_–P_75_), days]	7.50 (4.00–10.00)
Hospitalization days [M(P_25_–P_75_), days]	6.00 (5.00–8.00)
Bronchoscopy time [M(P_25_–P_75_), days]	11.00 (9.00–13.00)
Pulmonary manifestations	
Hypoxemia [*N*(%)]	33 (34.38)
Atelectasis [*N*(%)]	17 (17.71)
Pleural effusion [*N*(%)]	12 (12.50)
Lung necrosis [*N*(%)]	1 (1.04)
Extrapulmonary manifestations	
Digestive system [*N*(%)]	6 (6.25)
Toxic encephalopathy [*N*(%)]	6 (6.25)
Skin manifestations [*N*(%)]	5 (5.21)
Circulatory system [*N*(%)]	2 (2.08)
Arthritis [*N*(%)]	1 (1.04)
Hematologic system [*N*(%)]	0 (0)
Urinary system [*N*(%)]	0 (0)

*Note*: Non‐normal data were expressed as median (interquartile range), and normal data were expressed as mean ± standard deviation. Digestive system manifestations included abdominal discomfort (3 cases) and liver enzymes more than double the normal reference value (three cases); skin manifestations included nonspecific rash (four cases) and urticaria (one case); circulatory system manifestations included cardiac enzymes more than double the normal reference value (one case) and frequent premature ventricular contractions (one case).

Abbreviations: BALF, bronchoalveolar lavage fluid; MPP, *Mycoplasma pneumoniae* pneumonia.

**Table 2:** Laboratory findings of 96 children with MPP.

	MPP children (*n* = 96)
WBC [M(P_25_–P_75_), *10^9^/L]	7.50 (6.28–9.79)
RBC [$\bar{x}$ ±s, *10^12^/L]	4.52 ± 0.47
HGB [$\bar{x}$ ±s, g/L]	123.56 ± 11.22
PLT [$\bar{x}$ ±s, *10^9^/L]	406.63 ± 152.19
CRP [M(P_25_–P_75_), mg/L]	4.00 (4.00–14.50)
PCT [M(P_25_–P_75_), ng/mL]	0.10 (0.06–0.20)
ALB [M(P_25_–P_75_), g/L]	43.25 (40.92–44.98)
ALT [M(P_25_–P_75_), U/L]	18.00 (14.00–22.75)
AST [M(P_25_–P_75_), U/L]	34.50 (27.00–41.15)
LDH [M(P_25_–P_75_), U/L]	329.00 (285.75–411.75)
Urea $\left[\bar{x}\pm s,\text{mmol}/L\right]$	3.13 ± 1.11
Crea [M(P_25_–P_75_), µmol/L]	27.00 (23.00–34.75)
FIB [M(P_25_–P_75_), g/L]	3.93 (3.05–4.50)
D‐D [M(P_25_–P_75_), mg/L)]	0.61 (0.42–1.61)
TnI‐Ultra [M(P_25_–P_75_), µg/L)]	0.00 (0.00–0.00)
Myoglobin [M(P_25_–P_75_), µg/L)]	18.49 (10.95–26.25)
CKMB [M(P_25_–P_75_), µg/L)]	0.80 (0.37–1.42)
BNP [M(P_25_–P_75_), pg/mL)]	10.93 (5.19–13.55)
BALF cytokines	
IL‐2[M(P_25_–P_75_), pg/mL]	1.25 (1.25–2.75)
IL‐4[M(P_25_–P_75_), pg/mL]	1.25 (1.25–1.25)
IL‐6[M(P_25_–P_75_), pg/mL]	159.72 (49.75–367.59)
IL‐10[M(P_25_–P_75_), pg/mL]	5.12 (1.25–13.87)
IL‐17A [M(P_25_–P_75_), pg/mL]	4.18 (1.25–14.41)
TNF‐α[M(P_25_–P_75_), pg/mL]	7.85 (1.25–24.68)
IFN‐γ[M(P_25_–P_75_), pg/mL]	6.21 (1.25–28.04)
BALF cytology	
Inflammatory cell count [M(P_25_–P_75_), *10^6^/L]	2535.00 (1150.00–4950.00)
Neutrophil [M(P_25_–P_75_), *10^6^/L]	973.00 (363.25–2501.43)
Lymphocyte [M(P_25_–P_75_), *10^6^/L]	423.90 (157.05–841.77)
Macrophage [M(P_25_–P_75_), *10^6^/L]	476.00 (219.60–941.18)
Epithelium [M(P_25_–P_75_), *10^6^/L]	79.50 (30.98–189.30)
Neutrophil [M(P_25_–P_75_),%]	44.00 (27.00–64.00)
Lymphocyte [M(P_25_–P_75_),%]	19.00 (8.00–34.00)
Macrophage [M(P_25_–P_75_),%]	23.00 (10.00–34.00)
Epithelium [M(P_25_–P_75_),%]	4.00 (2.00–9.00)

*Note*: Non‐normal data were expressed as median (interquartile range), and normal data were expressed as mean ± standard deviation.

Abbreviations: ALB, albumin; ALT, alanine transferase; AST, aspartate transferase; BALF, bronchoalveolar lavage fluid; BNP, B‐type brain natriuretic peptide; CKMB, creatine kinase MB isoenzyme; CRP, C‐reactive protein; D‐D, d‐dimer; FIB, fibrinogen; HGB, hemoglobin; LDH, lactate dehydrogenase; MPP, *Mycoplasma pneumoniae* pneumonia; PCT, procalcitonin; PLT, platelet; RBC, red blood cell; WBC, white blood cell.

### 3.2 Cytokine levels and cytological findings in BALF of the Low MP‐DNA MPP and High MP‐DNA MPP groups

Twenty‐eight and 68 children were enrolled in the Low MP‐DNA MPP and High MP‐DNA MPP groups, respectively. As shown in Tables 3 and 4, compared with that in the Low MP‐DNA MPP group, bronchoscopy was performed earlier; fever and hospitalization days were longer; C‐reactive protein (CRP), procalcitonin (PCT), fibrinogen (FIB), and d‐dimer (D‐D) levels were higher; and platelets (PLT), albumin (ALB), creatine kinase MB isoenzyme (CKMB) levels were lower in the High MP‐DNA MPP group (all *p* < .05).

**Table 3:** Patient characteristics, pulmonary and extrapulmonary manifestations of the Low MP‐DNA MPP and High MP‐DNA MPP groups.

	Low MP‐DNA MPP group	High MP‐DNA MPP group	Test	*p*‐value
	(*n* = 28)	(*n* = 68)		
Male [*N*(%)]	17 (60.71)	34 (50.00)	0.914	.375
Age [$\bar{x}$ ±s, years]	4.76 ± 2.85	5.61 ± 2.66	−1.386	.169
Weight [M(P_25_–P_75_), kg]	17.00 (11.50–22.13)	18.50 (15.00–23.38)	−1.650	.099
Fever days [M(P_25_–P_75_), days]	4.50 (0.00–10.00)	8.00 (5.25–10.75)	−2.445	.014
Hospitalization days [M(P_25_–P_75_), days]	6.00 (3.25–7.75)	7.00 (5.00–8.00)	−1.998	.046
Bronchoscopy time [M(P_25_–P_75_), days]	12.50 (10.25–15.00)	11.00 (9.00–12.75)	−2.570	.010
Pulmonary manifestations				
Hypoxemia [*N*(%)]	7 (25.00)	26 (38.24)	0.126	.215
Atelectasis [*N*(%)]	4 (14.29)	13 (19.12)	/	.770^a^
Pleural effusion [*N*(%)]	4 (14.29)	8 (11.76)	/	.742^a^
Lung necrosis [*N*(%)]	0 (0)	1 (1.47)	/	1.000^a^
Extrapulmonary manifestations				
Digestive system [*N*(%)]	0 (0)	6 (8.82)	/	.176^a^
Toxic encephalopathy [*N*(%)]	2 (7.14)	4 (5.88)	/	1.000^a^
Skin manifestations [*N*(%)]	0 (0)	5 (7.35)	/	.317^a^
Circulatory system [*N*(%)]	0 (0)	2 (2.94)	/	1.000^a^
Arthritis [*N*(%)]	0 (0)	1 (1.47)	/	1.000^a^
Hematologic system [*N*(%)]	0 (0)	0 (0)	/	/
Urinary system [*N*(%)]	0 (0)	0 (0)	/	/

*Note*: Non‐normal data were expressed as median (interquartile range), and normal data were expressed as mean ± standard deviation. Comparisons between the normally distributed parameters of the two groups were made with unpaired t‐tests. Mann‐Whitney *U* test was performed for nonparametric variables. Chi‐square test or Fisher's exact test was performed for classified variables.

Abbreviations: BALF, bronchoalveolar lavage fluid; MP, *Mycoplasma pneumoniae*; MPP, *Mycoplasma pneumoniae* pneumonia.

^a^ Fisher's exact test. Digestive system manifestations included abdominal discomfort (three cases) and liver enzymes more than double the normal reference value (three cases); skin manifestations included nonspecific rash (four cases) and urticaria (one case); circulatory system manifestations included cardiac enzymes more than double the normal reference value (one case) and frequent premature ventricular contractions (one case).

**Table 4:** Laboratory findings of the Low MP‐DNA MPP and High MP‐DNA MPP groups.

	Low MP‐DNA MPP group	High MP‐DNA MPP group	Test	*p*‐value
	(*n* = 28)	(*n* = 68)		
WBC [M(P_25_–P_75_), *10^9^/L]	7.66 (6.35–10.42)	7.45 (6.26–9.26)	−0.367	.714
RBC [$\bar{x}$ ±s, *10^12^/L]	4.54 ± 0.50	4.51 ± 0.46	0.206	.838
HGB [$\bar{x}$ ±s, g/L]	124.46 ± 11.78	123.19 ± 11.05	0.490	.626
PLT [$\bar{x}$ ±s, *10^9^/L]	486.50 ± 152.80	373.74 ± 140.24	3.488	.001
CRP [M(P_25_–P_75_), mg/L]	4.00 (4.00–4.00)	8.50 (4.00–18.88)	−2.845	.004
PCT [M(P_25_–P_75_), ng/mL]	0.07 (0.03–0.17)	0.11 (0.07–0.22)	−2.701	.007
ALB [M(P_25_–P_75_), g/L]	44.63 (42.40–46.20)	42.90 (39.68–44.60)	−2.515	.012
ALT [M(P_25_–P_75_), U/L]	18.00 (12.75–21.00)	18.00 (14.00–23.10)	−0.819	.413
AST [M(P_25_–P_75_), U/L]	31.50 (27.00–37.50)	36.00 (28.25–43.93)	−1.891	.059
LDH [M(P_25_–P_75_), U/L]	317.30 (263.48–396.75)	353.50 (413.75–291.25)	−1.233	.217
Urea [M(P_25_–P_75_), mmol/L]	3.45 (2.28–4.00)	2.95 (2.41–3.60)	−1.214	.225
Crea [M(P_25_–P_75_), µmol/L]	25.00 (21.25–29.80)	27.50 (23.18–35.00)	−1.605	.108
FIB [M(P_25_–P_75_), g/L]	2.93 (2.30–3.89)	4.11 (3.40–4.52)	−3.552	.001
D‐D [M(P_25_–P_75_), mg/L)	0.42 (0.26–0.61)	0.74 (0.50–1.83)	−3.426	.001
TnI‐Ultra [M(P_25_–P_75_), µg/L)]	0.00 (0.00–0.00)	0.00 (0.00–0.00)	−1.684	0.092
Myoglobin [M(P_25_–P_75_), µg/L)]	17.53 (10.94–26.10)	18.49 (10.95–26.25)	−0.222	.825
CKMB [M(P_25_–P_75_), µg/L)]	1.23 (0.79–1.77)	0.63 (0.25–1.20)	−3.146	.002
BNP [M(P_25_–P_75_), pg/mL)]	10.93 (5.46–15.24)	10.72 (4.97–13.33)	−0.048	.961

*Note*: Non‐normal data were expressed as median (interquartile range), and normal data were expressed as mean ± standard deviation. Comparisons between the normally distributed parameters of the two groups were made with unpaired *t*‐tests. Mann–Whitney *U* test was performed for nonparametric variables.

Abbreviations: ALB, albumin; ALT, alanine transferase; AST, aspartate transferase; BALF, bronchoalveolar lavage fluid; BNP, B‐type brain natriuretic peptide; CKMB, creatine kinase MB isoenzyme; CRP, C‐reactive protein; D‐D, d‐dimer; FIB, fibrinogen; HGB, hemoglobin; LDH, lactate dehydrogenase; MP, *Mycoplasma pneumoniae*; MPP, *Mycoplasma pneumoniae* pneumonia; PCT, procalcitonin; PLT, platelet; RBC,red blood cell; WBC, white blood cell.

**Figure 1:**
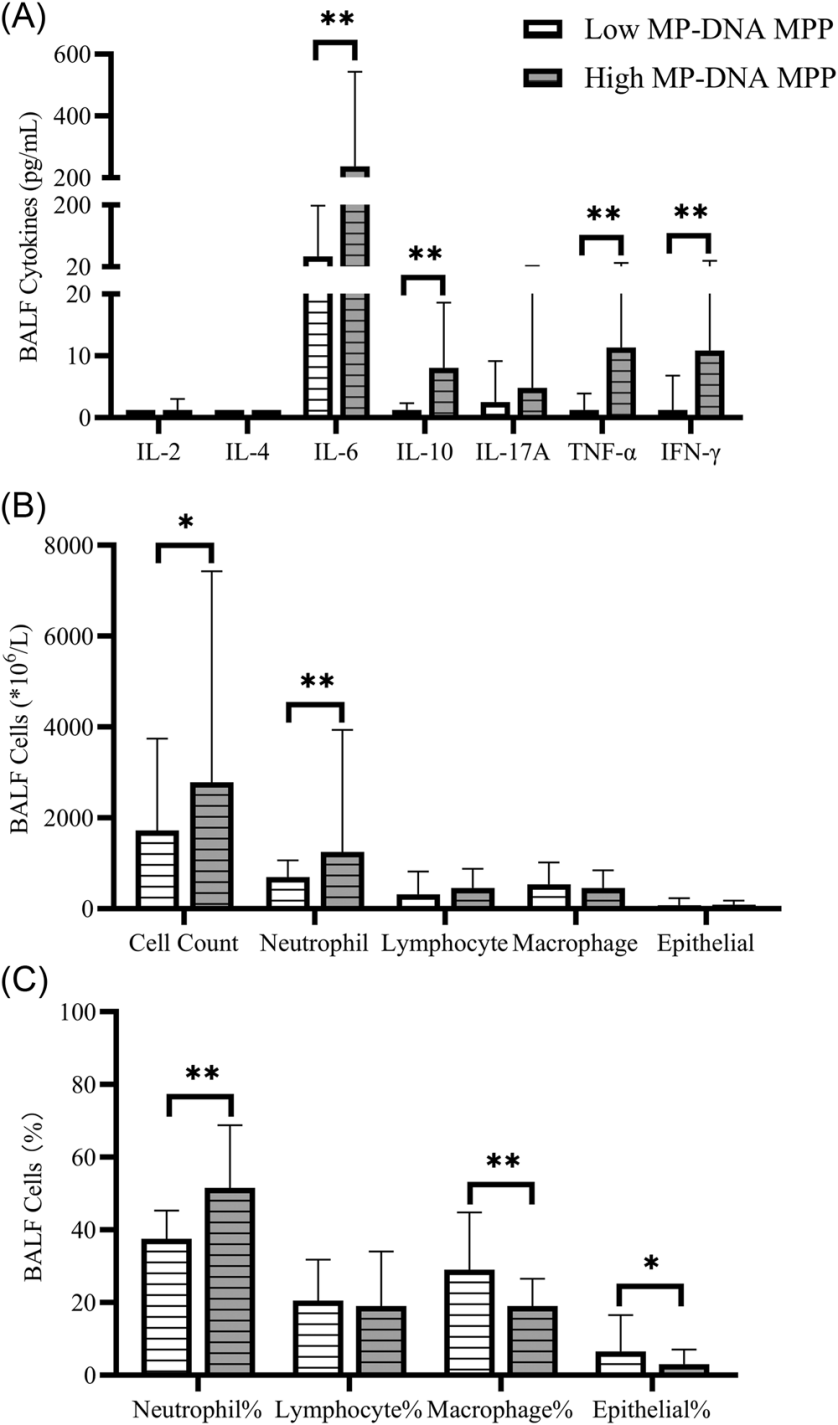
Cytokines and inflammatory cell levels in the BALF of the Low MP‐DNA MPP and High MP‐DNA MPP groups. (A) Cytokine level, (B) Inflammatory cell count, (C) Proportion of inflammatory cells. The plot represents the median and interquartile range. *p < .05, **p < .01, Low MP‐DNA MPP group versus High MP‐DNA MPP group. BALF, bronchoalveolar lavage fluid; MP, *Mycoplasma pneumoniae*; MPP, *Mycoplasma pneumoniae* pneumonia.

As shown in Figure 1, IL‐6 [236.60 (74.77–542.63) pg/mL versus 50.01 (10.45–197.51) pg/mL], IL‐10 [8.07 (3.70–18.60) pg/mL versus 1.25 (1.25–2.35) pg/mL], TNF‐α [11.37 (3.68–30.83) pg/mL versus 1.25 (1.25–3.91) pg/mL], and IFN‐γ [10.85 (3.05–37.06) pg/mL versus 1.25 (1.25–6.80) pg/mL] levels in the BALF of the High MP‐DNA MPP group increased significantly as compared with those in the Low MP‐DNA MPP group (all *p* < .05). The inflammatory cell count [2780.00 (1482.50–7427.50) × 10^6^/L versus 1722.50 (800.00–3742.50) × 10^6^/L] and neutrophil count [1253.00 (555.80–3933.45) × 10^6^/L versus 697.78 (105.30–1061.00) × 10^6^/L] in the BALF of the High MP‐DNA MPP group increased significantly as compared with that in the Low MP‐DNA MPP group (all *p* < .05). The neutrophil proportion [52.00 (30.00–69.00)% versus 37.56 (14.00–45.25)%] in the BALF of the High MP‐DNA MPP group was significantly higher than that of the Low MP‐DNA MPP group, while the macrophage proportion [19.00 (6.00–27.00)% versus 29.00 (20.75–44.75)%] and epithelial cell proportion [3.00 (1.00–7.00)% versus 6.50 (2.25–16.50)%] were significantly lower than those of the Low MP‐DNA MPP group (all *p* < .05).

### 3.3 Cytokine levels and cytological findings in BALF of the Non‐fever MPP and Fever MPP groups

The Non‐fever MPP and Fever MPP groups comprised 14 and 82 children, respectively. The Fever MPP group had a longer duration of hospitalization compared with the Non‐fever MPP group. It exhibited elevated levels of CRP, FIB, and D‐D but lower levels of TnI‐Ultra, as indicated in Tables 5 and 6. (all *p* < .05). The MP‐DNA level of the Fever MPP group was higher than that of the Non‐fever MPP group [10^7^ (10^6^–10^7^) copies/mL versus 10^5^ (10^4^–10^6^) copies/mL, *p* < .05]. The fever days in the Fever MPP group were 8.50 (6.00–11.00) days.

**Table 5:** Patient characteristics, pulmonary and extrapulmonary manifestations of the Non‐fever MPP and Fever MPP groups.

	Non‐fever MPP group (*n* = 14)	Fever MPP group (*n* = 82)	Test	*p*‐value
Male [*N*(%)]	7 (50)	44 (53.60)	0.064	.800
Age [$\bar{x}$ ±s, years]	5.15 ± 3.35	5.40 ± 2.63	−0.488	.626
Weight [M(P_25_–P_75_), kg]	17.00 (11.75–33.38)	18.00 (14.38–23.00)	−0.405	.685
Fever Days [M(P_25_–P_75_), days]	0 (0)	8.50 (6.00–11.00)	/	/
Hospitalization days [M(P_25_–P_75_), days]	3.50 (3.00–7.00)	7.00 (5.00–8.00)	−2.981	.003
Bronchoscopy time [M(P_25_–P_75_), days]	11.00 (8.75–13.49)	11.00 (9.00–13.00)	−0.151	.880
Pulmonary manifestations				
Hypoxemia [*N*(%)]	2 (14.29)	31 (37.80)	/	.128^a^
Atelectasis [*N*(%)]	2 (14.29)	15 (18.29)	/	1.000^a^
Pleural effusion [*N*(%)]	2 (14.29)	10 (12.20)	/	.686^a^
Lung necrosis [*N*(%)]	0 (0)	1 (1.22)	/	1.000^a^
Extrapulmonary manifestations				
Digestive system [*N*(%)]	0 (0)	6 (7.32)	/	.588^a^
Toxic encephalopathy [*N*(%)]	0 (0)	6 (7.32)	/	.588^a^
Skin manifestations [*N*(%)]	0 (0)	5 (6.10)	/	1.000^a^
Circulatory system [*N*(%)]	0 (0)	2 (2.44)	/	1.000^a^
Arthritis [*N*(%)]	0 (0)	1 (1.22)	/	1.000^a^
Hematologic system [*N*(%)]	0 (0)	0 (0)	/	/
Urinary system [*N*(%)]	0 (0)	0 (0)	/	/

*Note*: Non‐normal data were expressed as median (interquartile range), and normal data were expressed as mean ± standard deviation. Comparisons between the normally distributed parameters of the two groups were made with unpaired *t*‐tests. Mann–Whitney *U* test was performed for nonparametric variables. Chi‐square test or Fisher's exact test was performed for classified variables.

Abbreviations: BALF, bronchoalveolar lavage fluid; MPP, *Mycoplasma pneumoniae* pneumonia.

^a^ Fisher's exact test. Digestive system manifestations included abdominal discomfort (three cases) and liver enzymes more than double the normal reference value (three cases); skin manifestations included nonspecific rash (four cases) and urticaria (one case); circulatory system manifestations included cardiac enzymes more than double the normal reference value (one case) and frequent premature ventricular contractions (one case).

**Table 6:** Laboratory findings of the Non‐fever MPP and Fever MPP groups.

	Non‐fever MPP group (*n* = 14)	Fever MPP group (*n* = 82)	Test	*p*‐value
WBC [M(P_25_–P_75_), *10^9^/L]	7.13 (5.27–9.40)	7.57 (6.29–10.17)	−0.623	.527
RBC [$\bar{x}$ ±s, *10^12^/L]	4.44 ± 0.44	4.54 ± 0.48	−0.733	.465
HGB [$\bar{x}$ ±s, g/L]	123.36 ± 15.09	123.60 ± 10.54	−0.074	.941
PLT [$\bar{x}$ ±s, *10^9^/L]	420.64 ± 139.27	404.24 ± 154.95	0.371	.711
CRP [M(P_25_–P_75_), mg/L]	4.00 (4.00–4.00)	4.00 (4.00–17.25)	−2.232	.026
PCT [M(P_25_–P_75_), ng/mL]	0.09 (0.03–0.17)	0.10 (0.06–0.21)	−1.578	.114
ALB [M(P_25_–P_75_), g/L]	44.48 (42.43–44.75)	43.00 (40.85–45.43)	−0.794	.427
ALT [M(P_25_–P_75_), U/L]	18.33 (15.00–25.38)	18.00 (14.00–22.25)	−0.384	.701
AST [M(P_25_–P_75_), U/L]	32.50 (26.75–38.25)	34.50 (27.00–43.00)	−0.945	.345
LDH [M(P_25_–P_75_), U/L]	314.65 (269.93–348.00)	343.00 (287.25–412.00)	−1.043	.297
Urea [$\bar{x}$ ±s, mmol/L]	3.43 ± 1.88	3.08 ± 0.93	1.122	.265
Crea [M(P_25_–P_75_), µmol/L]	25.00 (20.98–30.00)	27.00 (23.53–35.00)	−1.688	.091
FIB [M(P_25_–P_75_), g/L]	2.67 (2.12–3.22)	4.05 (3.32–4.52)	−3.889	.001
D‐D [M(P_25_–P_75_), mg/L)]	0.33 (0.22–0.57)	0.67 (0.44–1.75)	−2.938	.003
TnI‐Ultra [M(P_25_–P_75_), µg/L)]	0.00 (0.00–0.00)	0.00 (0.00–0.00)	−2.575	.010
Myoglobin [M(P_25_–P_75_), µg/L)]	21.01 (11.82–28.20)	17.89 (10.89–26.25)	−0.415	.678
CKMB [M(P_25_–P_75_), µg/L)]	1.10 (0.68–1.96)	0.74 (0.36–1.43)	−1.029	.304
BNP [M(P_25_–P_75_), pg/mL)]	8.26 (4.50–12.42)	11.49 (5.26–14.34)	−0.874	.397

*Note*: Non‐normal data were expressed as median (interquartile range), and normal data were expressed as mean ± standard deviation. Comparisons between the normally distributed parameters of the two groups were made with unpaired *t*‐tests. Mann–Whitney *U* test was performed for nonparametric variables.

Abbreviations: ALB, albumin; ALT, alanine transferase; AST, aspartate transferase; BALF, bronchoalveolar lavage fluid; BNP, B‐type brain natriuretic peptide; CKMB, creatine kinase MB isoenzyme; CRP, C‐reactive protein; D‐D, d‐dimer; FIB, fibrinogen; HGB, hemoglobin; LDH, lactate dehydrogenase; MP, *Mycoplasma pneumoniae*; MPP, *Mycoplasma pneumoniae* pneumonia; PCT, procalcitonin; PLT, platelet; RBC,red blood cell; WBC, white blood cell.

**Figure 2:**
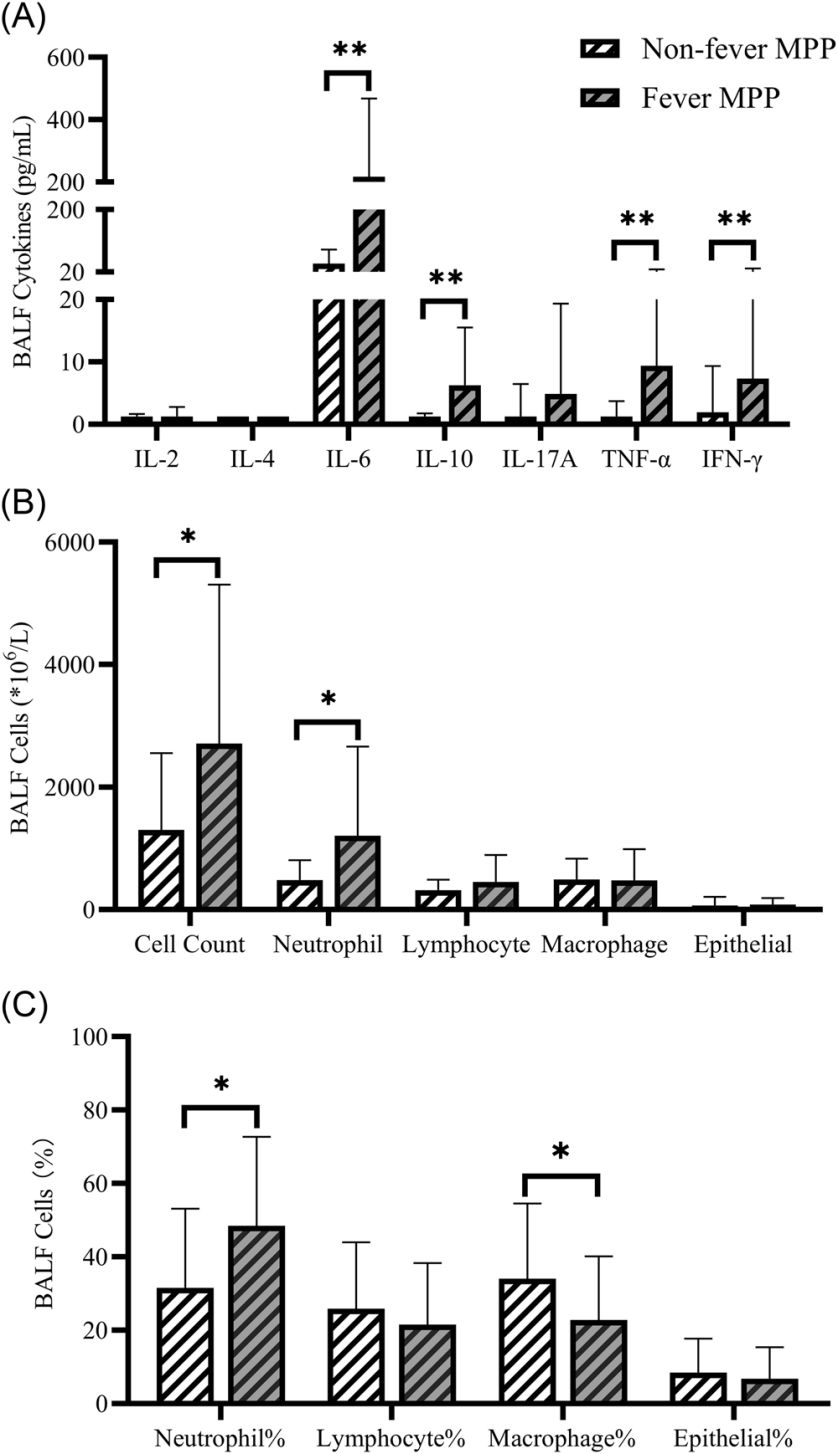
Cytokines and inflammatory cell levels in the BALF of the Non‐fever MPP and Fever MPP groups. (A) Cytokine level, (B) Inflammatory cell count, (C) Proportion of inflammatory cells. The plot represents the median and interquartile range. *p < .05, **p < .01, Non‐fever MPP group versus Fever MPP group. BALF, bronchoalveolar lavage fluid; MPP, *Mycoplasma pneumoniae* pneumonia.

As shown in Figure 2, IL‐6 [216.79 (71.86–467.94) pg/mL versus 43.92 (19.39–85.13) pg/mL], IL‐10 [6.27 (1.25–15.53) pg/mL versus 1.25 (1.25–1.72) pg/mL], TNF‐α [9.37 (1.25–27.26) pg/mL versus 1.25 (1.25–3.71) pg/mL], and IFN‐γ [7.33 (2.32–30.48) pg/mL versus 1.96 (1.25–9.35) pg/mL] levels in the BALF of the Fever MPP group increased significantly as compared to that in the Non‐fever MPP group (all *p* < .05). Inflammatory cell count [2705.00 (1392.50–5305.00) × 10^6^/L versus 1300.00 (832.50–2559.48) × 10^6^/L], neutrophil count [1205.20 (500.80–2658.25) × 10^6^/L versus 481.48 (96.30–809.00)  × 10^6^/L) and its proportion [48.00 (30.00–66.00)% versus 29.00 (13.50–44.29)%] in the BALF of the Fever MPP group were significantly higher than those of the Non‐fever MPP group, while the macrophage proportion [21.00 (7.00–31.00)% versus 31.40 (18.75–40.50)%] in the BALF of the Fever MPP was significantly lower than that of the Non‐fever MPP (all *p* < .05).

### 3.4 Cytokine levels and cytological findings in BALF of the Defervescence MPP and Fervescence MPP groups

The 82 children in the Fever MPP group were further subdivided into the Defervescence MPP group (*n* = 65) and the Fervescence MPP group (*n* = 17) based on the time of bronchoscopy. Tables 7 and 8 revealed that the Fervescence MPP group had an earlier bronchoscopy compared to the Defervescence MPP group. Additionally, the Fervescence MPP group exhibited a longer duration of fever and hospitalization, higher levels of CRP, PCT, alanine aminotransferase (ALT), aspartate transferase (AST), lactate dehydrogenase (LDH), FIB, and D‐D, and lower levels of red blood cell (RBC), PLT, and ALB (all *p* < .05). The MP‐DNA level of the Fervescence MPP group was higher than that of the Defervescence MPP group [10^8^ (10^7^–10^8^) copies/mL versus 10^6^ (10^5^–10^7^) copies/mL, *p* < .05].

**Table 7:** Patient characteristics, pulmonary and extrapulmonary manifestations of the Defervescence MPP and Fervescence MPP groups.

	Defervescence MPP group	Fervescence MPP group	Test	*p*‐value
	(*n* = 65)	(*n* = 17)		
Male [*N*(%)]	34 (52.31)	10 (58.82)	0.230	.631
Age [$\bar{x}$ ±s, years]	5.27 ± 2.82	5.92 ± 1.71	−1.419	.156
Weight [M(P_25_–P_75_), kg]	17.00 (13.75–23.00)	20.00 (17.50–22.25)	−1.477	.140
Fever Days [$\bar{x}$ ±s, days]	7.45 ± 3.32	11.29 ± 1.93	−4.201	.001
Hospitalization days [M(P_25_–P_75_), days]	6.00 (5.00–7.50)	8.00 (7.00–9.50)	−3.763	.001
Bronchoscopy time [M(P_25_–P_75_), days]	12.00 (9.50–14.00)	10.00 (8.50–11.00)	−2.801	.005
Pulmonary manifestations				
Hypoxemia [*N*(%)]	24 (36.92)	7 (41.18)	0.104	.747
Atelectasis [*N*(%)]	12 (18.46)	3 (17.65)	/	1.000^a^
Pleural effusion [*N*(%)]	7 (10.77)	3 (17.65)	/	.425^a^
Lung necrosis [*N*(%)]	1 (1.54)	0 (0)	/	1.000^a^
Extrapulmonary manifestations				
Digestive system [*N*(%)]	2 (3.08)	4 (23.53)	/	.015^a^
Toxic encephalopathy [*N*(%)]	5 (7.69)	1 (5.88)	/	1.000^a^
Skin manifestations [*N*(%)]	3 (4.62)	2 (11.76)	/	.275^a^
Circulatory system [*N*(%)]	1 (1.54)	2 (11.76)	/	.374^a^
Arthritis [*N*(%)]	1 (1.54)	0 (0)	/	1.000^a^
Hematologic system [*N*(%)]	0 (0)	0 (0)	/	/
Urinary system [*N*(%)]	0 (0)	0 (0)	/	/

*Note*: Non‐normal data were expressed as median (interquartile range), and normal data were expressed as mean ± standard deviation. Comparisons between the normally distributed parameters of the two groups were made with unpaired *t*‐tests. Mann–Whitney *U* test was performed for nonparametric variables. Chi‐square test or Fisher's exact test was performed for classified variables.

Abbreviations: BALF, bronchoalveolar lavage fluid; MPP, *Mycoplasma pneumoniae* pneumonia.

^a^ Fisher's exact test. Digestive system manifestations included abdominal discomfort (three cases) and liver enzymes more than double the normal reference value (three cases); skin manifestations included nonspecific rash (four cases) and urticaria (one case); circulatory system manifestations included cardiac enzymes more than double the normal reference value (one case) and frequent premature ventricular contractions (one case).

**Table 8:** Laboratory findings of the Defervescence MPP and Fervescence MPP groups.

	Defervescence MPP group	Fervescence MPP group	Test	*p*‐value
	(*n* = 65)	(*n* = 17)		
WBC [M(P_25_–P_75_), *10^9^/L]	7.66 (6.52–10.23)	6.89 (5.70–10.27)	−0.829	.407
RBC [$\bar{x}$ ±s, *10^12^/L]	4.59 ± 0.44	4.31 ± 0.56	2.245	.028
HGB [M(P_25_–P_75_), g/L]	124.00 (116.00–131.25)	126.00 (113.00–130.50)	−0.464	.643
PLT [M(P_25_–P_75_), *10^9^/L]	406.00 (309.00–513.50)	264.00 (235.50–464.00)	−2.597	.009
CRP [M(P_25_–P_75_), mg/L]	4.00 (4.00–12.00)	19.00 (4.00–41.50)	−2.999	.003
PCT [M(P_25_–P_75_), ng/mL]	0.09 (0.55–0.20)	0.19 (0.13–0.66)	−2.728	.006
ALB [M(P_25_–P_75_), g/L]	43.30 (41.25–45.55)	41.10 (36.50–43.55)	−2.586	.010
ALT [M(P_25_–P_75_), U/L]	17.00 (13.00–21.00)	19.00 (17.00–40.15)	−1.993	.046
AST [M(P_25_–P_75_), U/L]	32.20 (26.50–40.50)	43.00 (33.50–57.25)	−3.199	.001
LDH [M(P_25_–P_75_), U/L]	324.00 (266.00–393.50)	442.00 (376.00–633.50)	−3.861	.001
Urea [$\bar{x}$ ±s, mmol/L]	3.13 ± 0.96	2.89 ± 0.77	0.930	.355
Crea [M(P_25_–P_75_), µmol/L]	27.00 (23.00–34.50)	31.00 (26.45–35.00)	−1.311	.190
FIB [$\bar{x}$ ±s, g/L]	3.99 ± 1.19	4.23 ± 1.09	−0.492	.623
D‐D [M(P_25_–P_75_), mg/L)]	0.60 (0.43–1.30)	1.78 (0.85–2.26)	−2.791	.005
TnI‐Ultra [M(P_25_–P_75_), µg/L)]	0.00 (0.00–0.00)	0.00 (0.00–0.00)	−0.299	.765
Myoglobin [M(P_25_–P_75_), µg/L)]	18.34 (11.25–25.95)	17.44 (9.64–29.01)	−0.017	.986
CKMB [M(P_25_–P_75_), µg/L)]	0.81 (0.39–1.51)	0.52 (0.16–1.17)	−1.448	.148
BNP [M(P_25_–P_75_), pg/mL)]	11.86 (4.63–14.96)	10.38 (6.97–12.46)	−0.029	.977

*Note*: Non‐normal data were expressed as median (interquartile range), and normal data were expressed as mean ± standard deviation. Comparisons between the normally distributed parameters of the two groups were made with unpaired *t*‐tests. Mann–Whitney *U* test was performed for nonparametric variables.

Abbreviations: ALB, albumin; ALT, alanine transferase; AST, aspartate transferase; BALF, bronchoalveolar lavage fluid; BNP, B‐type brain natriuretic peptide; CKMB, creatine kinase MB isoenzyme; CRP, C‐reactive protein; D‐D, d‐dimer; FIB, fibrinogen; HGB, hemoglobin; LDH, lactate dehydrogenase; MP, Mycoplasma pneumoniae; MPP, Mycoplasma pneumoniae pneumonia; PCT, procalcitonin; PLT, platelet; RBC,red blood cell; WBC, white blood cell.

**Figure 3:**
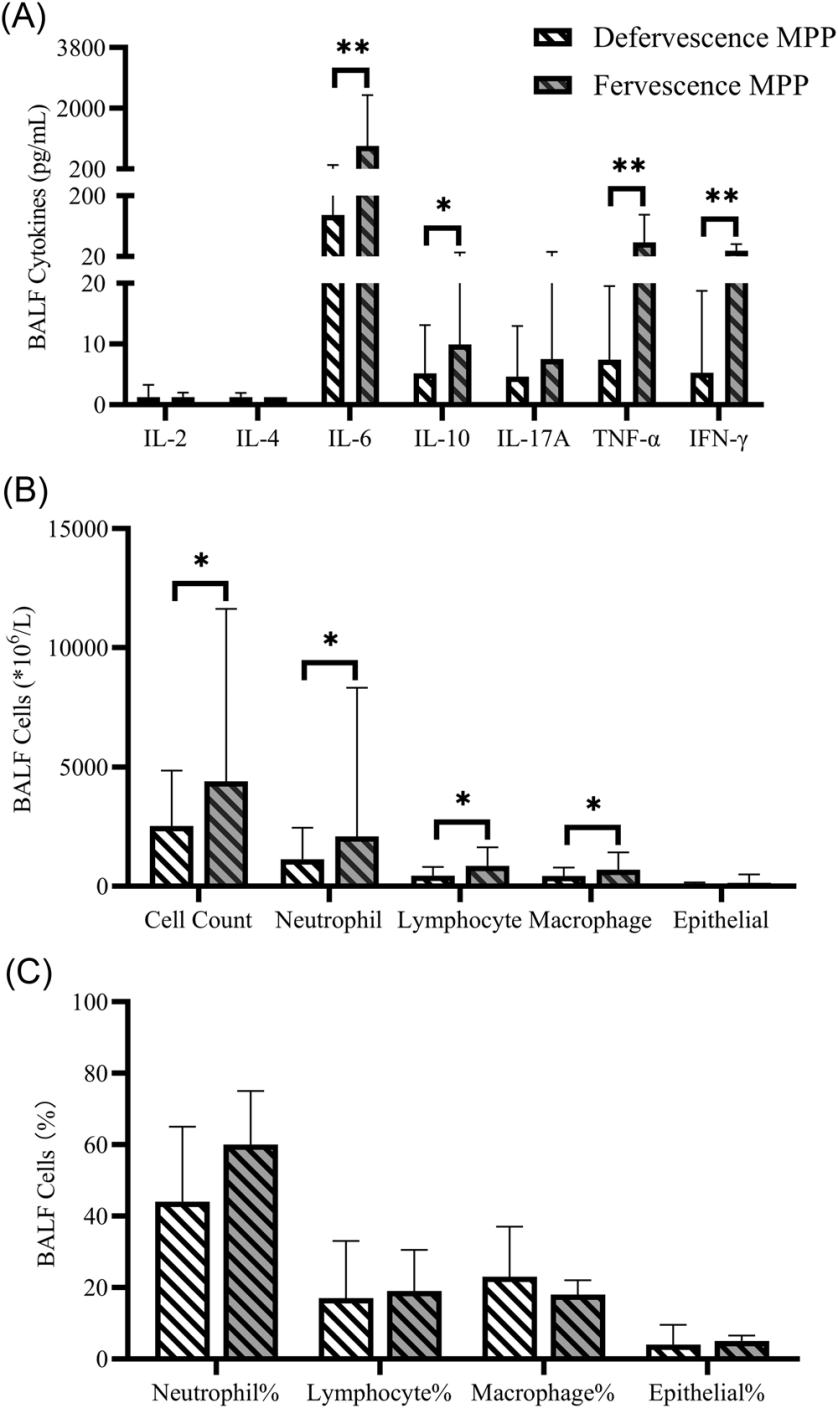
Cytokines and inflammatory cell levels in the BALF of the Defervescence MPP and Fervescence MPP groups. (A) Cytokine level, (B) Inflammatory cell count, (C) Proportion of inflammatory cells. The plot represents the median and interquartile range. *p < .05, **p < .01, Defervescence MPP group versus Fervescence MPP group. BALF, bronchoalveolar lavage fluid; MPP, *Mycoplasma pneumoniae* pneumonia.

As shown in Figure 3, IL‐6 [885.32 (243.94–2388.26) pg/mL versus 143.07 (54.94–317.36) pg/mL], IL‐10 [9.94 (5.90–31.99) pg/mL versus 5.16 (1.25–13.12) pg/mL], TNF‐α [62.32 (15.91–143.91) pg/mL versus 7.44 (1.25–18.77) pg/mL], and IFN‐γ [37.13 (13.43–56.33) pg/mL versus 5.25 (1.25–18.77) pg/mL] levels of the Fervescence MPP group were significantly increased as compared to those of the Defervescence MPP group (all *p* < .05). In addition, the inflammatory cell count [4400.00 (2385.00–11630.50) × 10^6^/L versus 2530.00 (1010.00–4860.00) × 10^6^/L], neutrophil count [2097.60 (691.90–8320.93) × 10^6^/L versus 1134.00 (357.35–2460.85) × 10^6^/L), macrophage count [698.00 (377.80–1412.63) × 10^6^/L versus 426.60 (185.40–781.70) × 10^6^/L], and lymphocyte count [855.60 (300.40–1633.70) × 10^6^/L versus 444.60 (136.40–812.40) × 10^6^/L] in the BALF of the Fervescence MPP group increased significantly as compared to those of the Defervescence MPP group (all *p* < .05).

### 3.5 Cytokine levels and cytological findings in BALF of the Mild MPP and Severe MPP groups

The Mild and Severe MPP groups had 37 and 59 children each, respectively. Bronchoscopy was performed later in the severe MPP group than in the mild MPP group; fever and hospitalization days were longer; CRP, PCT, AST, LDH, FIB, and D‐D levels were higher; and hemoglobin (HGB), ALB, and urea levels were lower, as shown in Tables 9 and 10. (all *p* < .05). There was no significant difference in MP‐DNA levels between the two groups (*p* > .05).

**Table 9:** Patient characteristics, pulmonary and extrapulmonary manifestations of the Mild MPP and Severe MPP groups.

	Mild MPP group (*n* = 37)	Severe MPP group (*n* = 59)	Test	*p*‐value
Male [*N*(%)]	18 (48.65)	33 (55.93)	0.484	.486
Age [$\bar{x}$ ±s, years]	5.77 ± 2.98	5.11 ± 2.55	−0.900	.368
Weight [M(P_25_–P_75_), kg]	18.00 (15.25–23.50)	17.00 (13.50–22.50)	−0.757	.449
Fever Days [M(P_25_–P_75_), days]	5.00 (0.50–8.50)	9.00 (6.00–12.00)	−3.282	.001
Hospitalization days [M(P_25_–P_75_), days]	6.00 (3.00–7.00)	7.00 (5.00–9.00)	−3.526	.001
Bronchoscopy time [M(P_25_–P_75_), days]	10.00 (8.00–12.50)	11.00 (10.00–14.00)	−2.123	.034
Pulmonary manifestations				
Hypoxemia [*N*(%)]	0 (0)	33 (55.93)	/	/
Atelectasis [*N*(%)]	0 (0)	17 (28.81)	/	/
Pleural effusion [*N*(%)]	0 (0)	12 (20.34)	/	/
Lung necrosis [*N*(%)]	0 (0)	1 (1.69)	/	/
Extrapulmonary manifestations				
Digestive system [*N*(%)]	0 (0)	6 (10.17)	/	.079^a^
Toxic encephalopathy [*N*(%)]	0 (0)	6 (10.17)	/	/
Skin manifestations [*N*(%)]	1 (2.70)	4 (6.78)	/	.646^a^
Circulatory system [*N*(%)]	1 (2.70)	1 (1.69)	/	1.000^a^
Arthritis [*N*(%)]	0 (0)	1 (1.69)	/	1.000^a^
Hematologic system [*N*(%)]	0 (0)	0 (0)	/	/
Urinary system [*N*(%)]	0 (0)	0 (0)	/	/

*Note*: Non‐normal data were expressed as median (interquartile range), and normal data were expressed as mean ± standard deviation. Comparisons between the normally distributed parameters of the two groups were made with unpaired *t*‐tests. Mann–Whitney *U* test was performed for nonparametric variables. Chi‐square test or Fisher's exact test was performed for classified variables.

Abbreviations: BALF, bronchoalveolar lavage fluid; MPP, *Mycoplasma pneumoniae* pneumonia.

^a^ Fisher's exact test. Digestive system manifestations included abdominal discomfort (three cases) and liver enzymes more than double the normal reference value (three cases); skin manifestations included nonspecific rash (four cases) and urticaria (one case); circulatory system manifestations included cardiac enzymes more than double the normal reference value (one case) and frequent premature ventricular contractions (one case).

**Table 10:** Laboratory findings of the Mild MPP and Severe MPP groups.

	Mild MPP group (*n* = 37)	Severe MPP group (*n* = 59)	Test	*p*‐value
WBC [M(P_25_–P_75_), *10^9^/L]	7.25 (6.18–9.56)	7.73 (6.50–10.42)	−0.621	.535
RBC [$\bar{x}$ ±s, *10^12^/L]	4.60 ± 0.38	4.47 ± 0.51	1.306	.195
HGB [$\bar{x}$ ±s, g/L]	127.89 ± 9.74	120.85 ± 11.31	3.130	.002
PLT [M(P_25_–P_75_), *10^9^/L]	418.00 (290.00–482.00)	385.00 (277.00–522.00)	−0.105	.916
CRP [M(P_25_–P_75_), mg/L]	4.00 (4.00–11.00)	4.00 (4.00–18.50)	−1.690	.091
PCT [M(P_25_–P_75_), ng/mL]	0.08 (0.04–0.17)	0.11 (0.07–0.21)	−2.090	.037
ALB [$\bar{x}$ ±s, g/L]	44.25 ± 2.67	41.61 ± 4.16	−3.031	.002
ALT [M(P_25_–P_75_), U/L]	17.00 (13.50–21.50)	18.00 (15.00–23.10)	−1.699	.089
AST [M(P_25_–P_75_), U/L]	32.00 (26.00–38.50)	36.00 (31.00–43.20)	−2.215	.027
LDH [M(P_25_–P_75_), U/L]	313.00 (272.95–382.50)	371.00 (291.00–443.00)	−2.003	.045
Urea [$\bar{x}$ ±s, mmol/L]	3.41 ± 1.20	2.95 ± 1.02	2.008	.048
Crea [$\bar{x}$ ±s, µmol/L]	29.31 ± 8.34	28.25 ± 7.65	0.641	.523
FIB [$\bar{x}$ ±s, g/L]	3.54 ± 1.21	4.05 ± 1.20	−2.003	.045
D‐D [M(P_25_–P_75_), mg/L)	0.54 (0.35–0.77)	0.71 (0.44–2.02)	−2.865	.004
TnI‐Ultra [M(P_25_–P_75_), µg/L)]	0.00 (0.00–0.00)	0.00 (0.00–0.00)	−0.147	.883
Myoglobin [M(P_25_–P_75_), µg/L)]	16.14 (13.65–25.95)	18.83 (9.31–27.18)	−0.440	.660
CKMB [M(P_25_–P_75_), µg/L)]	1.16 (0.53–1.92)	0.77 (0.28–1.26)	−1.778	.075
BNP [M(P_25_–P_75_), pg/mL)]	12.42 (4.66–15.75)	9.21 (5.15–12.49)	−1.040	.298

*Note*: Non‐normal data were expressed as median (interquartile range), and normal data were expressed as mean ± standard deviation. Comparisons between the normally distributed parameters of the two groups were made with unpaired *t*‐tests. Mann–Whitney *U* test was performed for nonparametric variables.

Abbreviations: ALB, albumin; ALT, alanine transferase; AST, aspartate transferase; BALF, bronchoalveolar lavage fluid; BNP, B‐type brain natriuretic peptide; CKMB, creatine kinase MB isoenzyme; CRP, C‐reactive protein; D‐D, d‐dimer; FIB, fibrinogen; HGB, hemoglobin; LDH, lactate dehydrogenase; MP, Mycoplasma pneumoniae; MPP, Mycoplasma pneumoniae pneumonia; PCT, procalcitonin; PLT, platelet; RBC,red blood cell; WBC, white blood cell.

**Figure 4:**
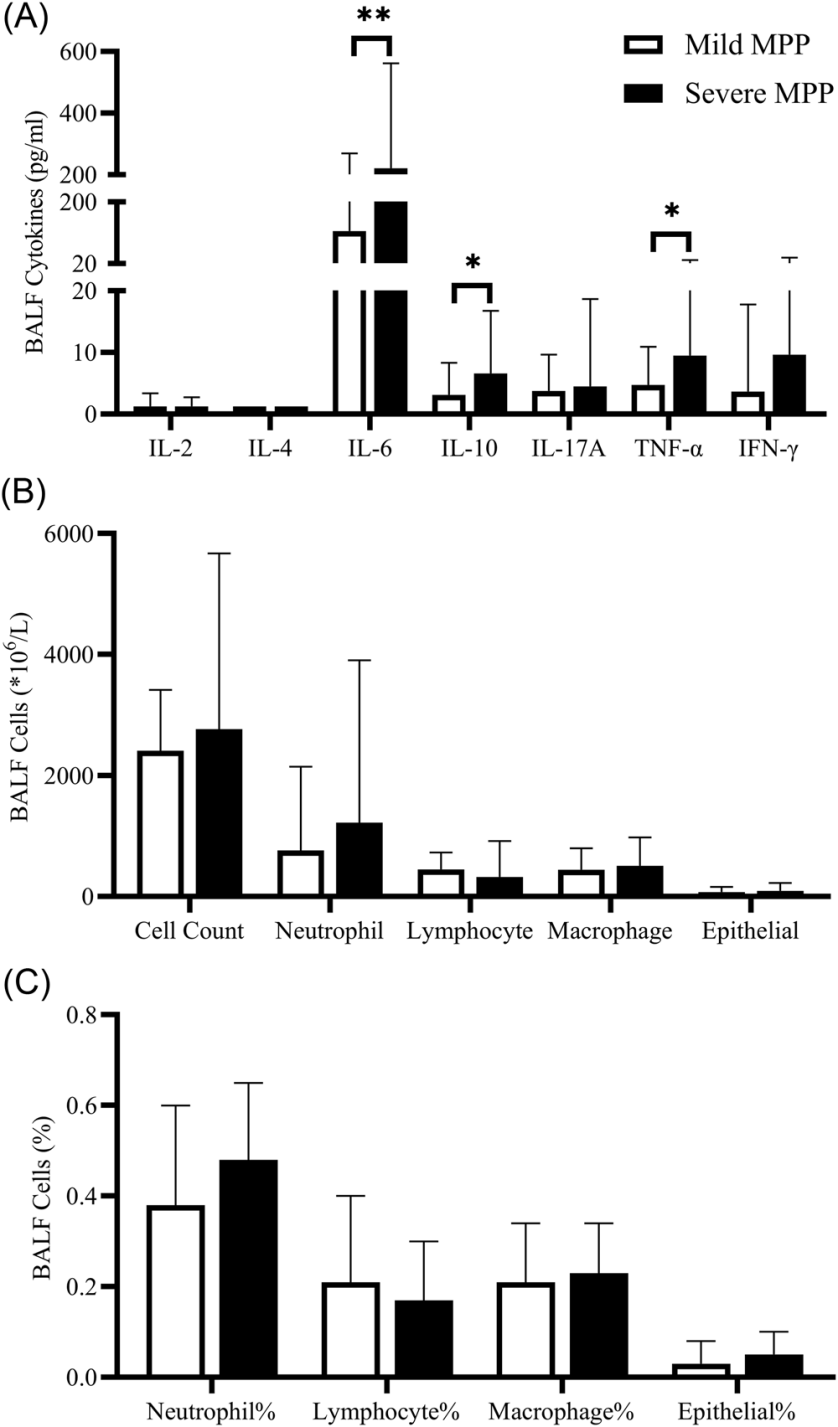
Cytokines and inflammatory cell levels in the BALF of the Mild MPP and Severe MPP groups. (A) Cytokine level, (B) Inflammatory cell count, (C) Proportion of inflammatory cells. The plot represents the median and interquartile range. *p < .05, **p < .01, Mild MPP group versus Severe MPP group. BALF, bronchoalveolar lavage fluid; MPP, *Mycoplasma pneumoniae* pneumonia.

As shown in Figure 4, IL‐6 [221.24 (73.59–561.74) pg/mL versus 114.70 (22.27–269.55) pg/mL], IL‐10 [6.59 (1.25–16.75) pg/mL versus 3.12 (1.25–8.33) pg/mL], and TNF‐α [9.50 (1.25–30.47) pg/mL versus 4.71 (1.25–10.89) pg/mL] levels in the BALF of the Severe MPP group increased significantly as compared to those in the Mild MPP group (all *p* < .05). The levels of inflammatory cells in the BALF were not significantly different between the two groups (*p* > .05).

### 3.6 Spearman's correlation analysis

**Figure 5:**
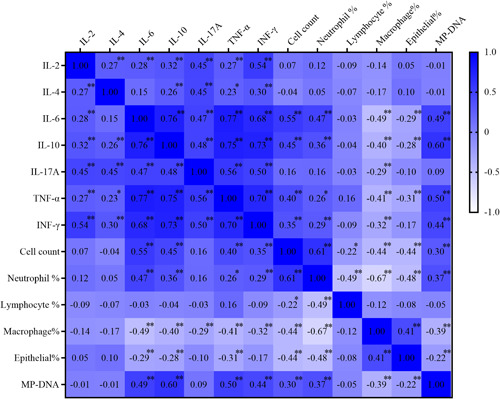
BALF parameter correlation heatmap from children with MPP. The bar represents the range of parameter correlations, ranging from –1 (white) to 1 (dark blue). The stronger the correlation, the greater the absolute value; the minus sign indicates a negative correlation. *p < .05, **p < .01. BALF, bronchoalveolar lavage fluid; MPP, *Mycoplasma pneumoniae* pneumonia; MP: *Mycoplasma pneumoniae*.

**Figure 6:**
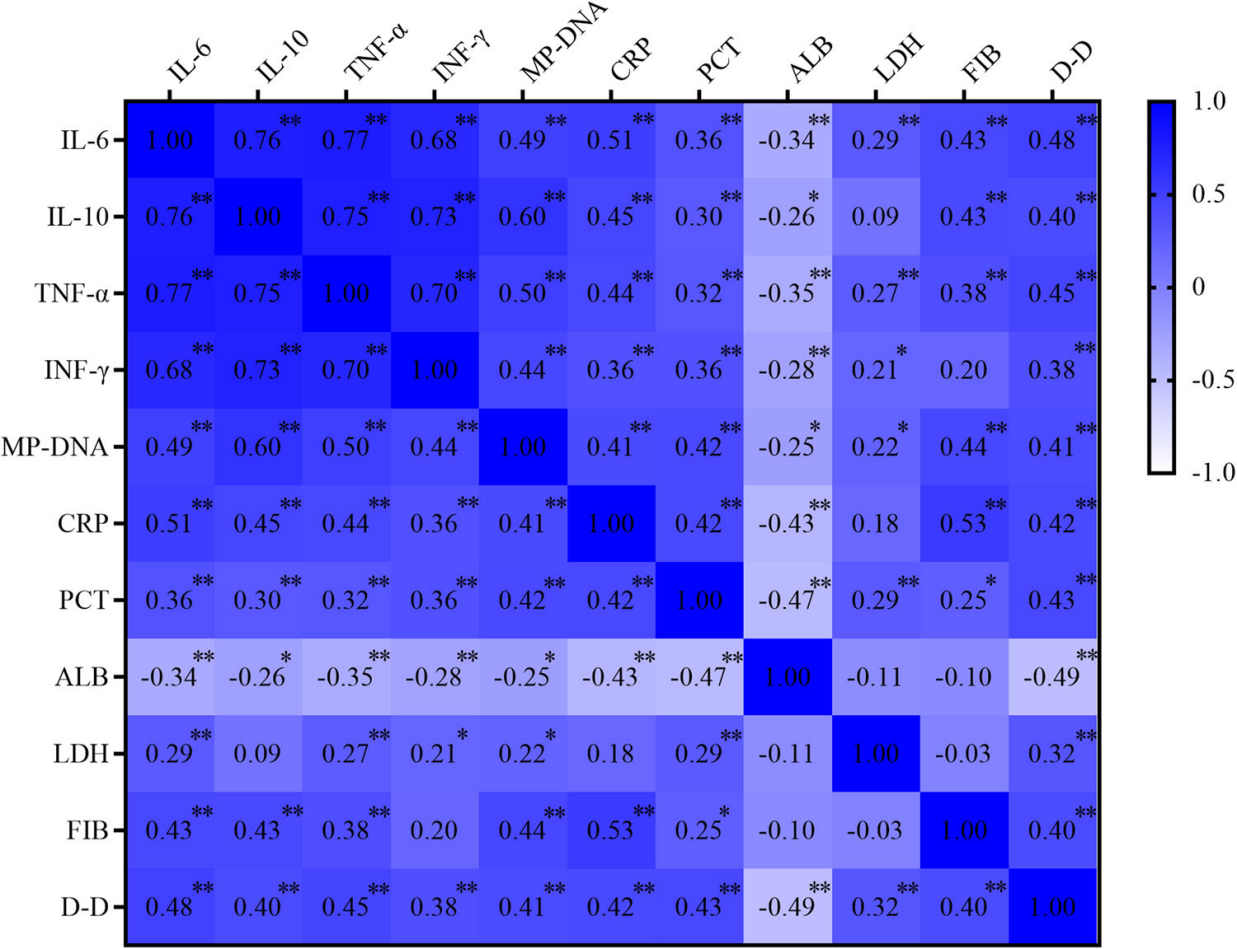
BALF and blood parameter correlation heatmap from children with MPP. The bar represents the range of parameter correlations, ranging from –1 (white) to 1 (dark blue). The stronger the correlation, the greater the absolute value; the minus sign indicates a negative correlation. *p < .05, **p < .01. ALB, albumin; BALF, bronchoalveolar lavage fluid; CRP, C‐reactive protein; D‐D, d‐dimer; FIB, fibrinogen; LDH, lactate dehydrogenase; MP, *Mycoplasma pneumoniae*; MPP, *Mycoplasma pneumoniae* pneumonia; PCT, procalcitonin.

To gain a deeper insight into the relationship between the parameters of pediatric patients with MPP, Spearman's correlation analysis was carried out (Figures 5 and 6). Figure 5 shows that in the BALF of children with MPP, the MP‐DNA load was positively correlated with IL‐6, IL‐10, TNF‐α, and IFN‐γ levels; inflammatory cell count; and neutrophil proportion, while it was negatively correlated with macrophage proportion (all *p* < .05). Figure 6 shows that the levels of IL‐6, IL‐10, TNF‐α, IFN‐γ, and MP‐DNA in the BALF of children with MPP were positively correlated with the levels of CRP, PCT, LDH, FIB, and D‐D but negatively correlated with the level of ALB (all *p* < .05).

## 4 DISCUSSION

In the present study, the High MP‐DNA MPP and Fervescence MPP groups had higher MP‐DNA levels in BALF; the High MP‐DNA MPP, Fervescence MPP, and Severe MPP groups had more days of fever; and the High MP‐DNA MPP, Fever MPP, Fervescence MPP, and Severe MPP groups had longer hospitalization days and stronger inflammatory immune response in their lungs. The BALF inflammatory response in children with MPP was characterized by an increase in the levels of IL‐6, IL‐10, TNF‐α, IFN‐γ, and neutrophils and a decrease in the proportion of macrophages. A correlation was identified between the pulmonary cytokine levels and the MP‐DNA load, as well as the serum inflammatory markers.

The children in the High MP‐DNA MPP, Fever MPP, Fervescence MPP, and Severe MPP groups showed increased levels of IL‐6, IL‐10, TNF‐α, and IFN‐γ in their BALF. The increased IFN‐γ level in the BALF of the Severe MPP group was, however, not significantly different from that of the Mild MPP group. Previous research has found that children with MPP and SMPP have significantly higher levels of IL‐6, IL‐10, TNF‐α, and IFN‐γ in their BALF.^13^, ^14^, ^15^ Elevated levels of IL‐6 and IL‐10 are indicative of the severity of inflammation and pneumonia.^16^ Activated alveolar macrophages and lymphocytes secrete IL‐6, IL‐10, and chemokines, causing neutrophils to migrate to infection sites.^17^ TNF‐α induces macrophages and neutrophils to release chemokines.^18^ Under the synergistic effect of IL‐17A and TNF‐α, neutrophils rapidly migrate to the inflammation site and persist.^19^ IFN‐γ can activate itself and nearby immune cells, which plays a vital role in the early host resistance to infection.^20^ The timely recruitment of neutrophils plays a crucial role in initiating effective host immune responses.^21^ Nevertheless, an excessive release of cytokines may result in the infiltration of neutrophils into the lungs, causing significant harm to target cells.^22^, ^23^

As an anti‐inflammatory cytokine, IL‐10 inhibits the effect of activated alveolar macrophages on T helper cells, reduces the release of pro‐inflammatory cytokines, inhibits neutrophil overproduction, and relieves the body's inflammatory response.^24^ The presence of elevated IL‐10 levels in the BALF of MPP children suggested that the body itself was controlling the inflammation. According to previous research, children with SMPP have lower serum IL‐10 levels than those with mild MPP.^25^ In our present study, the increase in IL‐10 level in the BALF of children with MPP was far less than the increase in IL‐6 level, indicating the relative insufficiency of anti‐inflammatory cytokines in the lungs. Thus, the imbalance of pro‐inflammatory and anti‐inflammatory effects was an important cause of lung injury in children with MPP, which was also supported by the findings of other studies.^26^

IL‐17A is a crucial factor for pulmonary protection, contributing significantly to the defense against infections and the preservation of epithelial cell homeostasis.^27^ Yang M et al. reported that the decreased IL‐17A level in the BALF of children with MPP is an indicator of disease severity.^28^ However, in our present study, IL‐17A level was somewhat elevated in children with MPP. Mikacenic C et al. found a significant correlation between IL‐17A and protein levels in the BALF of adult patients with acute respiratory distress syndrome; the authors also found that elevated IL‐17A levels led to increased alveolar permeability.^29^ During an infection or injury, the protective role of IL‐17A changes to a damaging role.^30^ IL‐17A has been linked to the pathogenesis of autoimmune diseases.^31^ The impact of IL‐17A on the body is intricate and may be contingent on both its concentration and the present state of the body. The levels of IL‐2 and IL‐4 in the BALF of pediatric patients with MPP were relatively low, indicating that their contribution to the local inflammatory response in the lungs of pediatric patients with MPP is minimal.

Children in the High MP‐DNA MPP and Fever MPP groups exhibited more prominent inflammatory cell infiltration in their lungs, with neutrophils being the predominant cells and a relatively lower proportion of macrophages. This finding suggests that MP infection leads to inflammation dominated by neutrophils. Previous studies have reported the infiltration of neutrophils in the BALF and serum of pediatric patients with RMPP.^32^ Neutrophils can change protein expression in the body and participate in the cytokine signaling pathway.^33^ The toxic inflammatory response caused by excessive amounts of cytokines results in the death of numerous macrophages, and the surviving macrophages also have varying degrees of functional impairment (immune paralysis).^34^ Macrophages play a crucial role in the clearance of MP from the lung, and damage to macrophages reduces the body's ability to clear MP.^35^ Thus, excessive neutrophil infiltration and impaired macrophage function resulting from MP infection promote the progression of local inflammation.

The MP‐DNA levels in the BALF of children with MPP were positively correlated with IL‐6, IL‐10, TNF‐α, and IFN‐γ levels; inflammatory cell count; and neutrophil proportion in the BALF and were negatively correlated with the macrophage proportion in the BALF. This finding suggests that there may be a dose–response relationship between the pathogen and the inflammatory immune response in the lungs. Chen Zhengrong et al. discovered that neutrophil infiltration was more significant in children with high MP loads than in children with low MP loads in their BALF.^36^ Fang et al.^37^ found that in children with SMPP, the higher the MP‐DNA copies in the BALF, the more severe the disease and the longer the duration of immunotherapy. However, no difference in the MP‐DNA load was observed in our present study between the Mild and Severe MPP groups. This finding suggests that the MP‐DNA load is not an absolute determinant of disease severity in children with MPP. The complete viral genome was not found in pathological tissues or even in the blood of some patients with severe COVID‐19.^38^ Therefore, in these cases, the pathogen was not the direct cause of target cell injury. Because the secondary pathological immune response may cause immunoinflammatory injury in these patients, immunotherapy is an important treatment option.

Our study found that IL‐6, IL‐10, TNF‐α, IFN‐γ, and MP‐DNA levels in the BALF were positively correlated with CRP, PCT, LDH, FIB, and D‐D levels in the blood and negatively correlated with the ALB level in the blood. CRP, PCT, and LDH are important biomarkers of inflammation, and their levels are positively correlated with the severity of inflammation.^39^ FIB and D‐D levels reflect the pathological role of the fibrinolytic and coagulation systems in lung injury.^39^ The decline in ALB levels may be attributed to increased vascular permeability caused by inflammation. The weak correlation between the BALF and blood parameters suggests that blood parameters might partly reflect lung inflammation. Moreover, in this study, children in the High MP‐DNA MPP, Fever MPP, and Severe MPP groups had a higher incidence of pulmonary or extrapulmonary complications. Although the pathogenesis of MP‐associated extrapulmonary complications is still not fully known, it has been reported that the pathogenesis of MP‐associated extrapulmonary complications includes injury caused directly by pathogens and activation of the immune system triggered by an inflammatory response.^3^ Furthermore, the overproduction of IgE and IL‐17A is also an important pathogenesis of autoimmune diseases.^31^, ^40^ In the current study, there was some elevation of IL‐17A in the High MP‐DNA MPP, Fever MPP, and Severe MPP groups, but this elevation was not statistically different, possibly due to the small sample size. However, it is very unfortunate that serum immunoglobulin data were lacking in this study. Therefore, it is hoped that future studies will add more information in this area and provide more information on the immune mechanisms of MP‐associated complications.

A strong immunopathological response is an important pathogenetic feature in patients with fatal pneumonia ^41^ and multisystem inflammatory syndrome.^42^ Therefore, inhibiting the excessive immunoinflammatory response may be an important therapeutic strategy to improve the condition of children with MPP. In this study, immunotherapy was administered to nine children with MPP. Glucocorticoids, which possess potent immunomodulatory properties and are cost‐effective and easily obtainable, were utilized.^11^ Previous research has shown that corticosteroids, either alone or in combination, can shorten the disease course in children with MPP while causing no adverse effects.^43^, ^44^, ^45^ A recent meta‐analysis found that glucocorticoids improve the health of children with SMPP and RMPP.^44^, ^46^ Children in the High MP‐DNA MPP, Fever MPP, Fervescence MPP, and Severe MPP groups had a significant inflammatory immune response in their lungs in the current study. Therefore, immunomodulatory therapy should be initiated cautiously and actively in MPP children with fever, severe disease, or a high MP‐DNA load in the BALF.

The present study has several limitations. A control group of healthy children could not be established because of the ethical limitations related to BALF sample collection from healthy children and the difficulty in determining whether relatively healthy children were completely free of lung inflammation. In this study, the confirmation of MP infection in pediatric patients with pneumonia was established through the detection of MP genes and MP serum‐specific antibodies. Clinical diagnoses of MPP without these confirmatory tests were not included in the study, and cases that lacked bronchoscopy and/or were unable to provide BALF for cytokine, cytological, and etiological testing were also excluded. Therefore, there was a selection bias. Finally, because the study was retrospective, the sample size was small. Additionally, our hospital did not perform some immunological parameters (total immunoglobulins, including IgE, and general lymphocyte immunophenotyping) as routine tests. Therefore, information about other immunological parameters related to MP infection could not be obtained, and we were unable to identify the immune mechanism by which complications occur in children with MPP. However, previous studies have pointed to elevated IgE as a possible immunopathogenic mechanism for complications following MP infection.^40^, ^47^, ^48^ We anticipate that our future studies will allow us to learn more about these topics.

## 5 CONCLUSION

In conclusion, our study demonstrated that in children with MPP, the High MP‐DNA MPP, Fever MPP, Fervescence MPP, and Severe MPP groups exhibited a more robust inflammatory immune response in the lungs. This pulmonary inflammatory response was characterized by an increase in the levels of IL‐6, IL‐10, TNF‐α, IFN‐γ, and neutrophils and a decrease in macrophage proportion. Meanwhile, the relationship between pulmonary cytokine levels, MP‐DNA load, and serum inflammatory parameters was found to be weak. These findings may offer clinical support for the use of immunotherapy in pediatric patients with MPP.

## AUTHOR CONTRIBUTIONS

**Fang Deng**: Conceptualization; data curation; formal analysis; investigation; methodology; project administration; supervision; writing—original draft; writing—review & editing. **Huiling Cao**: Methodology; software; writing—review & editing. **Xiaohua Liang**: Data curation; formal analysis; methodology; software. **Qubei Li**: Conceptualization; formal analysis; methodology; project administration; supervision. **Yang Yang**: Conceptualization; formal analysis; methodology; project administration; supervision. **Zhihua Zhao**: Project administration; software; supervision. **Junjie Tan**: Conceptualization; data curation; formal analysis; resources. **Guo Fu**: Conceptualization; data curation; formal analysis; resources. **Chang Shu**: Conceptualization; formal analysis; funding acquisition; methodology; project administration; supervision; writing—review & editing.

## CONFLICTS OF INTEREST STATEMENT

The authors declare no conflicts of interest.

## ETHICS STATEMENT

The present retrospective observational study was authorized by the Medical Research Ethics Committee of the Children's Hospital of Chongqing Medical University and registered at http://www.chictr.org.cn/with the registration number ChiCTR2000034048 (registration date: June 22, 2020). Data for this study were sourced from medical records and laboratory findings from prior clinical consultations. The requirement to obtain informed consent was waived.

## Supporting information

Supporting information.

## Data Availability

The datasets analyzed during the current study are available from the corresponding author upon reasonable request.
