# Supplementary material for: Analysis of cytokine levels, cytological findings, and MP‐DNA level in bronchoalveolar lavage fluid of children with Mycoplasma pneumoniae pneumonia
Source: Immun Inflamm Dis. 2023 May 8;11(5):e849. doi: 10.1002/iid3.849 (PMC10165957; doi:10.1002/iid3.849)
Supplement: Supplementary file 1 — Supporting information. [file IID3-11-e849-s001.docx]

**
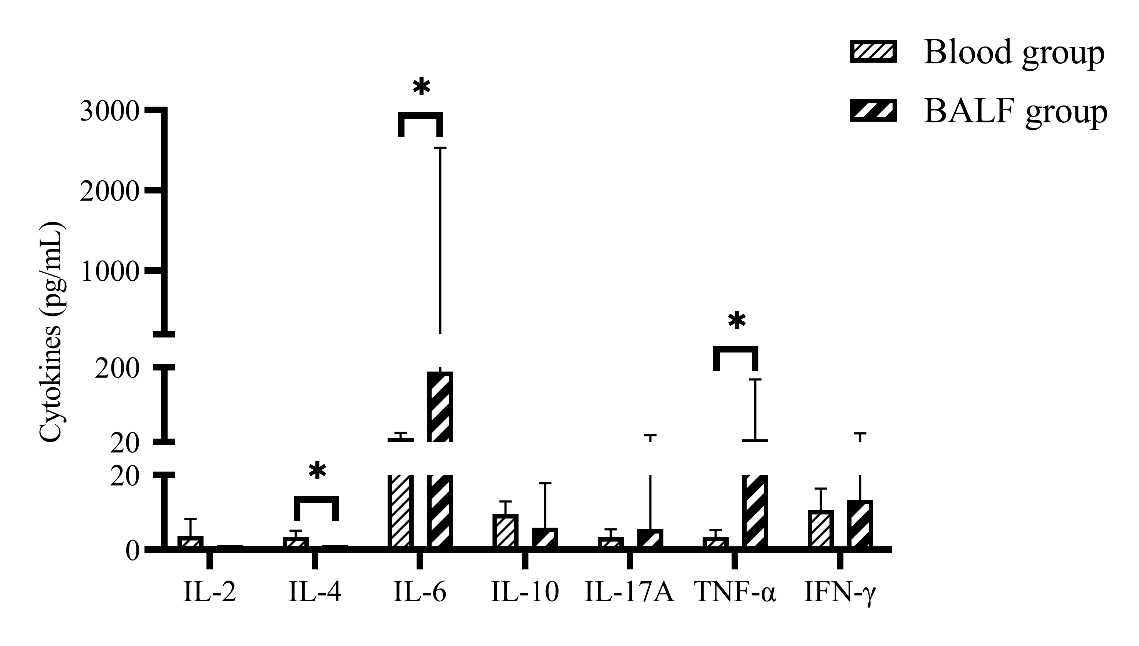
**

**Figure 1S** The levels of cytokines in the blood and in the BALF of 8 children with MPP. *P<0.05, blood group vs. BALF group. BALF: bronchoalveolar lavage fluid, MPP: *Mycoplasma pneumoniae* pneumonia.

Note: In the retrospective study, only 8 children with MPP were determined for both blood and BALF cytokine levels. As shown in the figure 1S, in the children with MPP, the levels of IL-6 and TNF-α in the BALF were increased significantly as compared to those in the blood, and the levels of IL-4 in the blood were increased significantly as compared to those in the BALF.

**Table 1S** Evaluation of the severity of pneumonia in children.

|  | Mild | Severe |
| --- | --- | --- |
| *General situation* | Good | Pale, depression, poor response |
| *Disorder of consciousness* | No | Yes |
| *Hypoxemia* | No | Cyanosis, tachypnea, grunting, nasal flaring, retractions (suprasternal, intercostals or subcostal), apnea, pulse oximetry measurement<92% |
| *Fever* | Inadequate severity | Fever over 41°C, persistent fever over 39°C>5 days |
| *Dehydration* | No | Decreased in body weight>3%, skin elasticity poor, mucous membranes dry, tears decreased, oliguria (urine volume <1ml/kg.h^-1^) |
| *Chest X-ray / CT* | Inadequate severity | Pleural effusion, pneumothorax, atelectasis, lung necrosis, lung abscess |
| *Extrapulmonary complications* | No | Sepsis, septic shock, toxic encephalopathy, hemolytic uremic syndrome |

Note: If any of the assessment items for severe pneumonia are met, the child has severe pneumonia; if none of the assessment items for severe pneumonia are met, the child has mild pneumonia. The evaluation of the severity of pneumonia in children are based on the British Thoracic Society guidelines for the management of community acquired pneumonia in children ^[1]^ and Guideline for diagnosis and treatment of community-acquired pneumonia in Children (2019 version) ^[2]^.

**References:**

1. Michael H, Julia C, Nicky C, Penny F, Anthony H, Michael M, et al. British Thoracic Society guidelines for the management of community acquired pneumonia in children: update 2011. THORAX 2011**:** i1-i23. DOI: 10.1136/thoraxjnl-2011-200598

2. National Health Commission of The People's Republic of China SAOT. Guideline for diagnosis and treatment of community-acquired pneumonia in Children (2019 version), vol. 12. Chin J Chin Infect Dis, 2019. DOI: 10.3760/cma.j.issn.1674-2397.2019.01.002
